# Ethylene signaling in roots: insights into gene regulatory networks

**DOI:** 10.1093/plphys/kiag240

**Published:** 2026-04-24

**Authors:** Blanca Jazmin Reyes-Hernández

**Affiliations:** Assistant Features Editor, Plant Physiology, American Society of Plant Biologists; Faculty of Science, Department of Plant and Environmental Sciences, Section for Plant Glycobiology, University of Copenhagen, Frederiksberg C 1871, Denmark

Ethylene is one of the simplest plant hormones, a 2-carbon gas, yet it controls remarkably complex developmental programs. In the early 20th century, its biological activity was recognized, and its effects in etiolated eudicot seedlings became known as the “triple response”: shorter roots and hypocotyls, thicker hypocotyls, and exaggerated apical hooks (Kieber and Schaller 2019). Since then, extensive research has identified major components of the ethylene signaling pathway (Binder 2020). However, important questions remain about how ethylene-responsive gene regulatory networks are organized across tissues and developmental contexts, especially in roots (Khoury et al. 2024).

Ethylene is perceived by membrane receptors located at the endoplasmic reticulum. In *Arabidopsis*, 5 ethylene receptors have been described: Ethylene Response (ETR)1, ETR2, Ethylene Response Sensor (ERS)1, ERS2, and Ethylene Insensitive 4 (EIN4). These receptors act as negative regulators: in the absence of ethylene, these receptors maintain the signaling pathway in an inactive state. Upon ethylene, this repression is relieved, leading to activation of the signaling pathway (Bakshi et al. 2015).

When the ethylene pathway is inactive, ethylene receptors activate the protein kinase CONSTITUTIVE TRIPLE RESPONSE 1 (CTR1), which suppresses downstream signaling through EIN2 phosphorylation and proteolysis. This allows EIN3-BINDING F-BOX (EBF) 1 and EBF2 to target ETHYLENE INSENSITIVE3 (EIN3) and EIN3-like (EIL) transcription factors (TFs), thereby causing their degradation (An et al. 2010). When the pathway is active, increased EIN2 represses EBF1/EBF2, stabilizing EIN3 and EIL and allowing signaling to proceed (Alonso et al. 1999). Beyond this EIN2-mediated effect, CTR1 may also contribute through its translocation to the nucleus, where it inhibits EBF1/EBF2, further stabilizing EIN3/EIL independent of its kinase activity (Park et al. 2023). In turn, these TFs activate secondary regulators, including ethylene response factors, and ethylene response DNA-binding factors, producing genome-wide transcriptional responses (Fig. 1) (Dolgikh, Pukhovaya, and Zemlyanskaya 2019).

**Figure 1:**
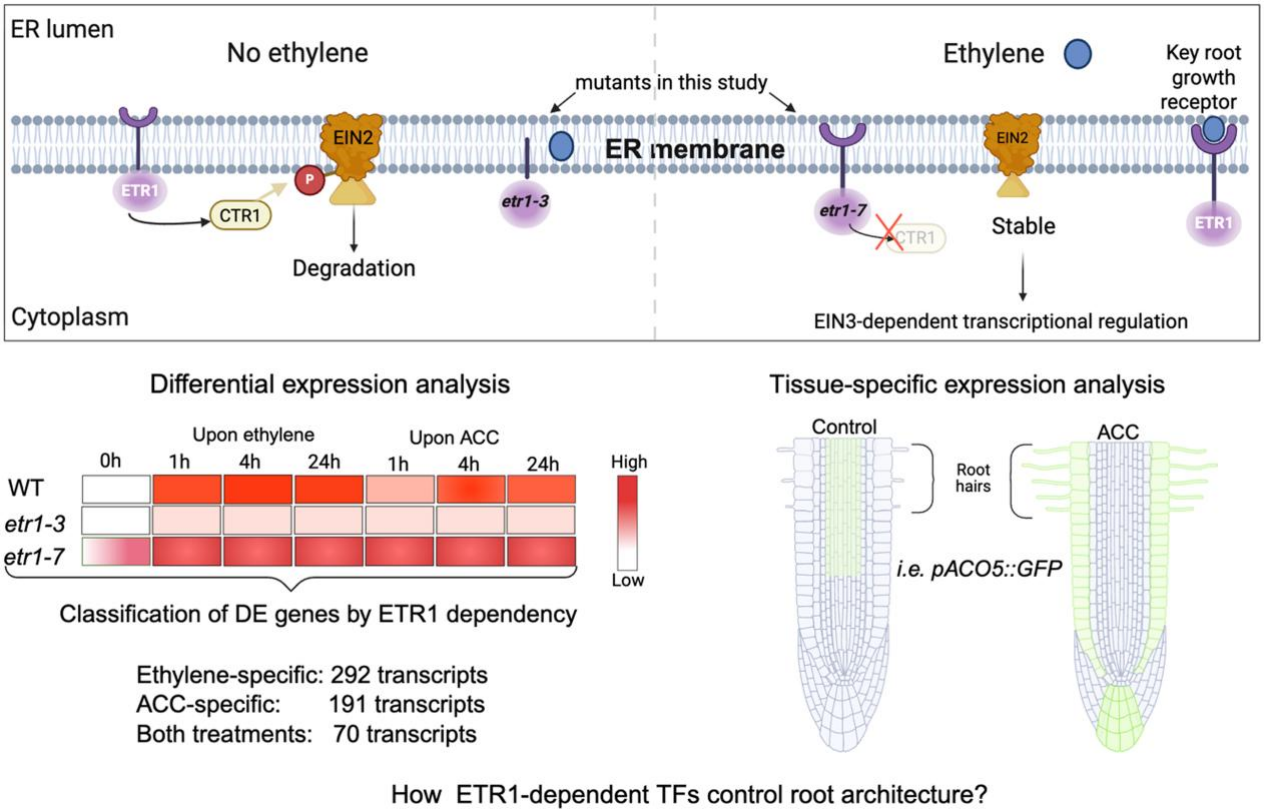
ETR1- and EIN3-dependent gene regulatory networks in roots (based on White et al. 2026). A model of ethylene signaling shown above. In the absence of ethylene (left), the ethylene receptors, including ETHYLENE RESPONSE1 (ETR1), activate the protein kinase CONSTITUTIVE TRIPLE RESPONSE1 (CTR1). CTR1 then phosphorylates ETHYLENE INSENSITIVE2 (EIN2), promoting its ubiquitination and degradation of EIN2. Low EIN2 levels also allow targeting the EIN3 transcription factor for degradation, preventing transcriptional regulation of ethylene signaling. In the presence of ethylene (right), the receptors bind ethylene, which is proposed to reduce CTR1 kinase activity, allowing EIN2 to accumulate. One role of EIN2 is to increase EIN3 stability. Additionally, upon ethylene perception, CTR1 may translocate to the nucleus and also contribute to further stabilizing EIN3 independently of CTR1 kinase activity. These combined effects lead to broad transcriptional changes. In White et al. (2026), ETR1-dependent transcriptional changes were investigated using 0.3 ppm ethylene and 0.75 µM 1-aminocyclopropane-1-carboxylic acid (ACC), after confirming that both treatments produced comparable responses in wild-type (WT) plants. White and colleagues performed RNA-Seq on roots and compared responses across different genotypes, including WT and 2 ethylene receptor mutants: *etr1-3*, which shows little response to ethylene or ACC, and *etr1-7*, which behaves similarly to ethylene-treated WT even without treatment. In WT plants, ethylene triggered a stronger and faster wave of gene expression changes than ACC. Differential expression (DE) analysis of their data led to the strict classification of 553 ETR1-dependent genes. Within this group, a subset was further classified into 292 ethylene-specific transcripts, 191 ACC-specific transcripts, and 70 transcripts responsive to both treatments. Notably, some ETR1-dependent genes included ACC OXIDASE (ACO), which catalyzes the conversion of ACC into ethylene. The authors demonstrated that ACC-to-ethylene conversion may be locally controlled in a cell-type–specific manner and may depend on the canonical ETR1–EIN3 signaling pathway. Together, this study provides a comprehensive dataset for investigating gene regulatory networks that control root development through both ETR1-dependent and ETR1-independent mechanisms. Created in https://BioRender.com.

Ethylene strongly shapes root architecture, and the ETR1 receptor is central to the resulting developmental outputs, responding not only to ethylene itself but also on its immediate precursor, 1-aminocyclopropane-1-carboxylic acid (ACC) (Khoury et al. 2024; Mou et al. 2025). Loss- and gain-of-function (*LOF* and *GOF*, respectively) studies indicate that ETR1 strongly influences primary root growth, lateral root formation, and root hair development (Harkey et al. 2018). Genetic studies also indicate that EIN2 is required for many root responses, while EIN3 and EIL1 are important TFs in root hair regulation. However, some ethylene-related root responses persist in *ein3* and *eil* mutants, suggesting that additional TFs and parallel regulatory modules are involved (Khoury et al. 2024). Although many ethylene-responsive genes in roots are known, the gene regulatory network (GRN) downstream of the ETR1 receptor has still not clearly been resolved. Defining ETR1- and EIN3-dependent GRNs is therefore an important next step to understand how ethylene signaling is translated into root developmental programs.

A recent study by White et al., (2026) published in *Plant Physiology* sheds light on this issue, showing how the ETR1 and EIN3-dependent GRNs respond to elevated levels of ACC and ethylene in Arabidopsis roots. The authors performed an RNA-Seq on roots of light-grown seedlings and compared responses across different genotypes, including wild-type Col-0 and 2 ethylene receptor mutants *etr1-3* (*GOF*) and *etr1-7* (*LOF)*.

To identify the ETR1-dependent GRN involved in ethylene- and ACC-mediated root development, the authors first established equivalent ethylene and ACC doses using root hairs increase in wild-type plants as a developmental readout. They found that 0.3 ppm ethylene produced a root hair response comparable to 0.75 µM ACC. Using these matching treatments, they tested ETR1 dependence using *etr1-3*, which barely responds to ethylene and ACC, and the *etr1-7* that already makes extra root hairs even without ethylene treatments. Together, these comparisons support a central role for ETR1 in mediating root hair responses to both ethylene and ACC.

Using these matched ACC and ethylene treatments, the authors next profiled gene expression dynamics in roots (Fig. 1). They performed RNA-Seq across wild-type and the *etr1-3* and *etr1-7* mutant lines, sampling 4 time points (0, 1, 4, and 24 h after treatment). These time points capture early, middle, and late transcriptional waves linked to root hair development. Principal component analysis showed clear genotype- and time-dependent shifts in global gene expression.

One striking result was that *etr1-7* samples clustered far from the others, fitting the idea that this line behaves as if ethylene signaling is partly active even without treatment. Supporting this, one late ethylene-treated wild-type sample began to shift toward the *etr1-7* expression pattern. The analysis also indicated that ACC and ethylene still produced distinguishable transcriptional profiles rather than fully overlapping. Overall, both ETR1 mutant lines showed a weaker transcriptional response than the wild type: *etr1-3* changed very little at early time points, while *etr1-7* already appeared partly “pretreated” at time zero. Together, these reinforce that ETR1 strongly controls the magnitude and timing of root transcriptional responses to ACC and ethylene.

Ethylene caused a stronger and faster wave of gene expression changes than ACC in wild-type plants. In both treatments, the response came in 2 clear “waves”: many genes going up and many genes going down. But ethylene affected more genes, and the shifts were bigger than with ACC, especially at the 1-hour mark. By 4 and 24 hours, both treatments showed more genes going down than up. Importantly, ACC and ethylene almost never pushed genes in opposite directions. Only a tiny number of genes flipped, meaning that the 2 signals mostly point the transcriptome the same way, just with different strength and timing. One interesting timing clue was that some shared genes responded more strongly to ACC at 4 hours, which fits the idea that ACC may need time to be converted into ethylene inside the plant (Fig. 1).

When the authors looked at what these genes code for, the pattern matched with root growth biology. Genes that went up were enriched for negative feedback on ethylene signaling and for auxin responses, suggesting that the plant is both controlling ethylene signaling and coordinating it with other hormones. Genes that went down were enriched for cell wall building and remodeling (like lignin and wall-loosening pathways), consistent with roots needing to loosen and remodel their walls during growth, lateral root emergence, and root hair formation. They also looked closely at the smaller set of genes that responded only to ACC (and not ethylene), since those could point to ACC-specific effects. That ACC-only group included signaling genes (including MAP kinase-related components), cell wall enzymes, and root hair regulators, suggesting that ACC might sometimes leave its own fingerprint. Alternatively, ACC may be converted into ethylene in specific cells, and the observed ACC-only group may have been masked when all cells were treated with ethylene.

Next, they tested how much of this transcriptional response depends on the ethylene receptor ETR1 by comparing the wild type to the 2 ETR1 mutants. Across the full dataset, most genes still moved in the same direction for ACC and ethylene, and ethylene usually triggered the strongest response. But the mutants revealed how ETR1 shapes the output. In the ethylene-insensitive *etr1-3* mutant, the response was clearly weaker, genes slightly shifted. In the *etr1-7* (“constitutive-signaling”) mutant, many genes looked “preshifted” even without treatment, and adding ACC or ethylene often caused little extra changes. Baseline comparisons made this especially clear: untreated wild type and *etr1-3* (GOF) looked very similar, while untreated *etr1-7* (LOF) already showed an enriched classic ethylene response.

To turn these transcriptional response patterns into a clean gene list, the authors set strict rules for calling a transcript “ETR1-dependent.” A gene had to respond in Col-0, fail to respond properly in *etr1-3* (GOF), and appear constitutively shifted in *etr1-7* (LOF) with little additional treatment effect. Using this filter, they identified 553 ETR1-dependent transcripts (Fig. 1). They also defined an “ETR1-independent” set, which were the genes that still responded similarly across genotypes and found a consistent shared response there too.

One interesting mechanistic insight came from ethylene biosynthesis genes. The authors focused on ACC OXIDASE (*ACO*) genes, which encode enzymes that convert ACC into ethylene. Several ACO transcripts increased after treatment, and unlike most genes, ACOs often responded more strongly to ACC than to ethylene. That suggests a feed-forward loop: supplying ACC may prompt the plant to build more of the machinery that turns ACC into ethylene, potentially amplifying ethylene production over time. They then tested this directly with RT-qPCR and showed that ACC-induced increases in ACO1, ACO2, and ACO4 depend on EIN3 and EIL1, the classic transcription factors in the canonical ethylene pathway, meaning this “amplification” still runs through the standard signaling chain.

Finally, the authors connected the observed gene changes to specific root tissues. Using cell-type datasets (Brady et al. 2007) and previously reported promoter reporters (Houben et al. 2024), they showed that different *ACO* genes were active in different root zones. For example, *ACO5* was enriched in root hair cells, and its reporter signal matched this tissue-specific distribution, which is particularly relevant given that ACC increases root hair number and length. Reflecting also localized expression, other ACO reporters were expressed in distinct tissues (root cap/columella, inner tissues, and lateral root primordia), suggesting that conversion of ACC to ethylene may be locally controlled in specific cell types rather than uniformly across cell types (Fig. 1).

To connect upstream signaling to downstream developmental outputs, the authors pulled TFs from the ETR1-dependent list, grouped them by shared response patterns, and identified which were direct targets of EIN3 using public datasets. They found 60 ETR1-dependent TFs transcripts and validated a subset of them by RT-qPCR in *ein3 eil1*. Among these transcripts, ROOT HAIR DEFECTIVE 6-LIKE 5 (RSL5), a TF linked to root hair development, was notable because its ACC-induced expression disappeared in *ein3 eil1*. These results helped delineate a clear regulatory cascade, linking ETR1 to EIN3, downstream TFs such as RSL5, and ultimately to root developmental programs.

Building on this approach, they also examined the ethylene- and ACC-responsive TF LATERAL ROOT PRIMORDIUM 1 (LRP1), known to function in lateral root development, to determine whether it displayed localized expression during root formation and whether its activity was modulated by ACC. Using a *proLRP1::GUS* reporter, they found that ACC strongly induced LRP1 expression in lateral root primordia, across multiple developmental stages and in emerged lateral roots, with prominent signals in central tissues such as the stele. The results mapped the ACC response to the sites where lateral roots are formed and matured.

However, *lrp1* mutant alleles showed only mild increase in lateral root emergence, and one allele also reduced primary root growth, compared to the wild type under control conditions. Upon ACC, lateral root emergence and primary root growth was strongly inhibited in the mutants, similar to wild type. Together, these data suggest that LRP1 contributes to the ACC-mediated inhibition of lateral root emergence and, to a limited extent, to the inhibition of the primary root growth. However, it is not essential on its own, indicating that other TFs downstream of ETR1-EIN3 can fulfill similar roles and highlighting functional redundancy within this regulatory network.

To further explore how ETR1-dependent TFs influence root development, they examined mutants for several additional TFs, including RAP2.6L, ANAC058, MYB9, and MYB52. Most observed mutant phenotypes were subtle, consistent with overlapping functions among TF families. Still, some effects were consistent and biologically meaningful: for example, *myb52-1* showed fewer root hairs and fewer lateral roots under certain conditions, and several mutants changed their sensitivity to ethylene depending on light or dark growth. These results support a “buffered” system where ACC/ethylene responses are robust, but individual TFs can fine-tune root traits, with their impact depending on developmental context.

Taken together, the elegant message from this work is clear: ACC and ethylene largely drive the same root transcriptional program, with ETR1 fine-tuning the intensity of the response. Downstream, a mix of genes, including ACO biosynthesis genes, tissue-specific expression patterns, and developmental TF networks, helps explain how hormone signaling turns into visible traits like root hairs, lateral roots emerged, and primary root growth. Notably, while LRP1 contributes to ACC-mediated inhibition of lateral root emergence, its potential role in the initiation of lateral root primordia offers an opportunity for future study. More broadly, this study raises a key questions for root architecture: which ETR1-dependent TFs, and in which cell types, control lateral root initiation vs emergence in response to ACC and ethylene? And how much of this control runs through EIN3-EIL1 vs other, noncanonical routes downstream of ETR1?

## Data Availability

No new data included in this article.
